# Henry A. Smith, A.M., D.D.S.

**Published:** 1913-12-15

**Authors:** Murdoch C. Smith, Waldo E. Boardman, Harry E. Cutter

**Affiliations:** Committee; Committee; Committee


### OBITUARY RESOLUTIONS.

### Henry A. Smith, A.M., D.D.S.

Henry A. Smith, an associate member of the American Academy of Dental Science, died at his home in Cincinnati, Ohio, September 10, 1913, at the ripe age of 82 years.

Born in Oxford, Ohio, February 28, 1832, he early manifested a disposition for the study of dentistry, and for more than fifty years he was one of the best-known men in his chosen calling.

Dr. Smith received the dental degree from the Ohio College of Dental Surgery in 1857 and the A. M. degree from the Miami University at Oxford in 1894.

For more than thirty years Dr. Smith served as dean of the Ohio College of Dental Surgery and during this time had become one of the leading authorities in his profession in the Middle West.

No man connected with the dental profession has done more to advance the research work in dental surgery. His efforts were crowned with distinction throughout the United States.

Not only did Dr. Smith devote the best years of his life to the imparting of his great skill and learning to others, but he was one of Cincinnati's leading practitioners, most of the time during his fifty years of professional life.

Dr. Smith became an Associate Fellow of the Academy in 1892 and it is fitting that this body should place upon its records its tribute to an eminent man—be it therefore,

*Resolved*, that in the death of Dr. Smith, the dental world has lost one of its most prominent, valued and respected members, and the Academy, an esteemed Associate Fellow, whose memory is forever engraved upon our records—a genial, wholesouled member.

*Resolved*, that a page be reserved for these minutes to be spread upon its records, and a copy be transmitted to the family of Dr. Smith.

MURDOCH C. SMITH,
WALDO E. BOARDMAN,
HARRY E. CUTTER,
*Committee.*

